# Juvenile Dermatomyositis in a Two-Year-Old Yemeni Girl in a Resource-Limited Setting: A Case Report

**DOI:** 10.7759/cureus.80376

**Published:** 2025-03-10

**Authors:** Bin-Sahel Omer, Naif Abduljabbar, Mohammed S Kutbi, Ahmed S Bani, Mohammed A Saghir

**Affiliations:** 1 Medicine and Surgery, Faculty of Medicine and Health Sciences, Seiyun University, Seiyun, YEM; 2 Community Health Nursing, Faculty of Nursing, University of Khartoum, Khartoum, SDN; 3 Preventive Medicine, Primary Health Care, Ministry of National Guards – Health Affairs, Jeddah, SAU; 4 Preventive Medicine, King Abdullah International Medical Research Center, Jeddah, SAU; 5 Preventive Medicine, King Saud Bin Abdul-Aziz University for Health Sciences, Jeddah, SAU; 6 Medicine and Surgery, Om Al-Qura Medical Centre, Seiyun, YEM; 7 Epidemiology, Graduate College, University of Bahri, Khartoum, SDN; 8 Faculty of Medicine, Cairo University, Cairo, EGY

**Keywords:** children disease, diagnostic delay, high fever, juvenil dermatomyositis, yemen

## Abstract

Juvenile dermatomyositis (JDM) is a rare systemic autoimmune vasculopathy primarily affecting children. It is characterized by muscular weakness and distinctive skin findings. This report describes the case of a two-year-old Yemeni girl from a resource-limited setting who presented with prolonged fever and later developed classic JDM symptoms, including malar rash, skin nodules, calcifications, and lower limb muscle weakness. Despite the unavailability of advanced diagnostic tools, the diagnosis was made based on clinical findings, elevated inflammatory markers, and muscle enzyme levels. Management included oral prednisolone, resulting in significant clinical improvement. This case highlights the challenges of diagnosing and managing atypical JDM presentations in resource-constrained areas while emphasizing the importance of clinical vigilance and multidisciplinary care. It also underscores the need for increasing awareness and better diagnostic access in low-resource settings. To our knowledge, this is the first reported case of JDM in Yemen.

## Introduction

Juvenile dermatomyositis (JDM) is a systemic inflammatory disorder that predominantly impacts the skin and muscles. This is the predominant kind of juvenile idiopathic inflammatory myopathy, exhibiting a yearly global incidence of two to four cases per million, influenced by ethnic and regional variables [1,2].

Typically, inflammatory proximal muscle weakness and characteristic skin lesions establish the diagnosis of JDM [3]. Nonetheless, individuals may also exhibit other symptoms such as tiredness, fever, arthritis, pain in the abdomen, gastrointestinal hemorrhage, and interstitial pneumonitis [4,5]. The disease exhibits a variable pattern, with recognized clinical patterns classified as monocyclic, polycyclic, or chronic-continuous based on clinical and laboratory remission within two years of diagnosis [4-6]. Advances in therapeutic approaches have significantly reduced mortality rates and improved functional outcomes in patients with JDM. Despite these advances, calcinosis remains a common long-term complication [7-9].

Various populations, including those in North India, Europe, Latin America, and Taiwan, have well-documented the clinical features of JDM [8,10-13]. Diagnosing JDM is particularly challenging in resource-limited settings, where access to adequate facilities, equipment, and expertise is constrained. Studies from Kenya (2016) [14], Morocco (2024) [15], and Nigeria (2011) [16] show how challenging it is to treat and diagnose JDM in these places, especially when unusual symptoms make things more complicated. Here, we present the case of a two-year-old Yemeni girl with atypical manifestations of JDM, managed in a resource-limited setting.

## Case presentation

A two-year-old Yemeni female from Hadramout was admitted to the pediatric ward of Om Al-Qura Medical Centre in Seiyun City with a history of fever persisting for more than two weeks, which occurs especially at night and is partially relieved by antipyretics, as reported by her family. She didn't have any complaints of vomiting, diarrhea, headaches, dysuria, or bone pain. Despite multiple courses of oral and intravenous antibiotics, there was no complete improvement.

On examination, the patient appeared ill, febrile (temperature: 38.4°C), mildly pale, with a pulse rate of 92 bpm and oxygen saturation of 97%. There was no cyanosis, jaundice, or organomegaly. The chest was clear with satisfactory air entry; the cardiovascular system (CVS) and abdomen examinations were normal.

**Figure 1:**
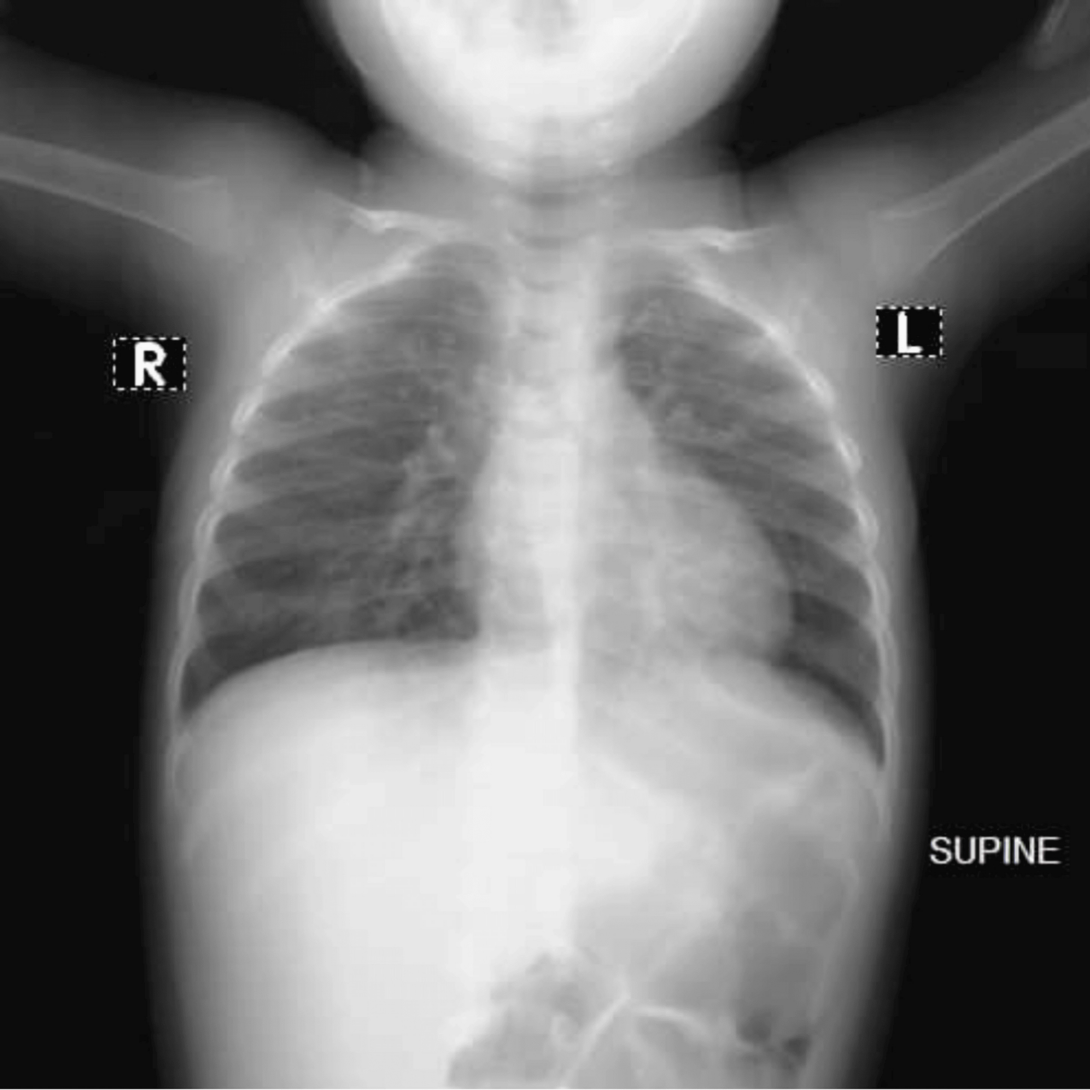
Normal AP supine chest X-ray AP: anteroposterior

Table 1 shows the patient had mild anemia (Hb=10.8 g/dL). Still, her WBC (9,000/mm³), random blood sugar (RBS) (112 mg/dL), cholesterol (175 mg/dL), lipase (46 u/L), creatine phosphokinase (CPK) (129 mcg/l), S. creatinine (0.4 mg/dl), rheumatoid factor (RF) (none reactive), antinuclear antibody (ANA) (negative), anti-ds DNA (10 IU/ml), and thyroid-stimulating hormone (TSH) (1.93 Uiu/ml), urine tests, tests for brucella, malaria, or dengue were normal. As well as ANA, C3 and C4 were normal. This person also had high platelets (622,000/L), erythrocyte sedimentation rate (ESR) (70 mm/1h), C-reactive protein (CRP) (positive), triglycerides (1138 mg/dL), aspartate aminotransferase (AST) (160 u/L), lactate dehydrogenase (LDH) (780 U/L), and alanine aminotransferase (ALT) (155 U/L). Imaging results included a normal chest X-ray (Figure 1) and a normal abdominal ultrasound. Bone marrow aspiration and biopsy were normal. We managed the patient for a fever of unknown origin (FUO) and treated him with parenteral ceftriaxone, antipyretics, and omega-3 for hyperlipidemia.

**Table 1:** Investigation during the first visit, after one month, and on follow-up N/A: this investigation is not done; WBC: white blood cells; HB: hemoglobin; ESR: erythrocyte sedimentation rate; CRP: C-reactive protein; RBS: random blood sugar; AST: aspartate aminotransferase; ALT: alanine aminotransferase; LDH: lactate dehydrogenase; CPK: creatine phosphokinase; RF: rheumatoid factor; ANA: antinuclear antibody; TSH: thyroid-stimulating hormone; mg/dL: milligrams (mg) per deciliter (dL); mm³: cubic millimeters; h: hour; L: liter; uIU/mL: micro-international units per milliliter; U/L: unit/liter

Investigation	Result of the first visit	Result after one month	Result on follow-up	Reference range
WBC	WBC 9000/mm³	13500/mm³	9500/mm³	4000-10000/mm³
HB	10.8 g/dL	12.3 g/dL	12.5 g/dL	11-16 g/dL
Platelets	622000/L	106700/L	350000/L	150000-400000/L
ESR	70 mm/1h	220 mm/1h	32 mm/1h	less than 15 mm/1 h
CRP	Positive 1/48	N/A	N/A	Negative < 6.0
RBS	112 mg/dl	N/A	90 mg/dl	Up to 180 mg/dl
Malaria	Negative	N/A	N/A	Negative
Dengue fever	Negative	N/A	N/A	Negative
Brucellosis	Negative	N/A	N/A	Negative
Triglycerides	1138 mg/dL	1424 mg/dl	197 mg/dl	Up to 200 mg/dl
Cholesterol	175 mg/dL	170 mg/dl	203 mg/dl	Up to 200 mg/dl
Uric acid	2.4 mg/dL	N/A	N/A	3.0-6.0 mg/dL
Lipase	46 u/L	46 u/L	40 u/L	Less than 60 u/L
AST	160 u/L	160 u/L	41 U/L	Less than 42 U/L
LDH	780 U/L	750 U/L	123U/L	Less than 160 U/L
ALT	155 U/L	150 U/L	40 U/L	Less than 40 U/L
CPK	129 mcg/l	125 mcg/l	115 mcg/l	10-120 mcg/l
S. creatinine	0.4 mg/dl	0.4 mg/dl	0.5 mg/dl	0.3-1.4 mg/dl
RF	Non-reactive	Non-reactive	Non-reactive	Up to 8IU/ML
ANA	Negative	N/A	N/A	Positive > 1/180, while Negative < 1/40
Anti-ds DNA	10 IU/ml	N/A	N/A	Non-reactive: less than 24, intermediate: 24-36, reactive: more than 36.0
TSH	1.93 Uiu/ML	N/A	N/A	.027-4.20 Uiu/ML

**Figure 2:**
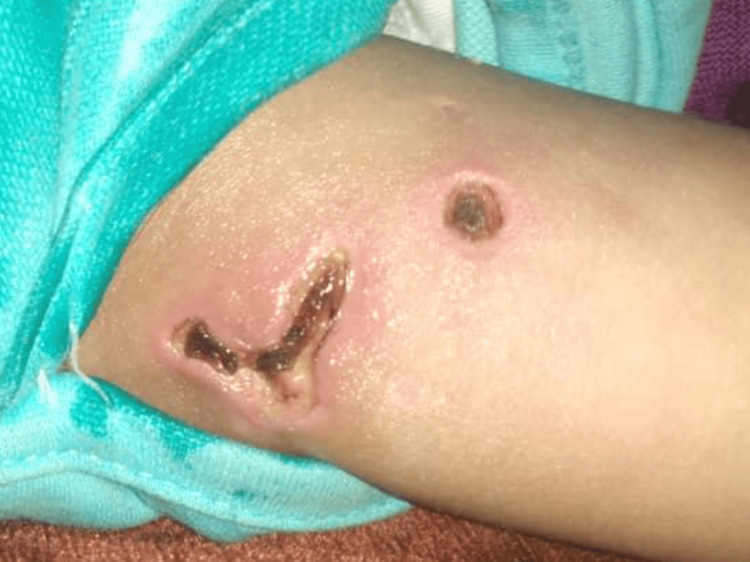
Skin nodules and areas of calcification on the left upper arm

One month later, the patient returned with new symptoms: skin rash over the face (malar and upper eyelids) and extremities. The patient presented with skin nodules and areas of calcification on the upper arms (Figure 2), lateral thighs, gluteal region, and abdomen, with some nodules displaying ulceration. The patient experienced bilateral lower limb weakness, tenderness, and wasting, resulting in an inability to stand or walk. On examination, the patient was conscious and febrile (temperature: 38.8°C, pulse: 94 bpm). The chest, CVS, and abdomen were normal. Lower limb examination showed muscle wasting, tenderness, and normal reflexes.

Investigations (Table 1) showed mild leukocytosis (WBCs = 13500/mm³), elevated inflammatory markers (ESR = 220 mm/1h), platelets, muscle enzymes, triglycerides, and cholesterol, while renal and liver function tests were normal. Electromyography (EMG) and a muscle biopsy were not performed due to unavailability and financial constraints. 

According to the diagnostic criteria for juvenile dermatomyositis established by Bohan and Peter [17], a diagnosis requires the presence of at least one characteristic skin rash, such as heliotrope rash, Shawl's sign, or Gottron's papules. Additionally, three of the following criteria must be met: symmetrical proximal muscle weakness, elevated muscle enzymes (including CK, LDH, ALT, AST, and aldolase), electromyographic changes indicative of myopathy, and muscle biopsy findings that show necrosis and inflammation. Consequently, this case was diagnosed as juvenile dermatomyositis based on these criteria.

The course of oral steroid therapy with prednisolone started at 2 mg/kg/day in divided doses for one month. After one month, the patient returned with significant clinical improvement, including the resolution of skin rash, nodules, and ulcerations. Additionally, the patient reported the disappearance of muscle tenderness and improved motor function, including the ability to stand and crawl. Additionally, the investigation showed a decline in ESR to 32 mm/h, normalized muscle enzymes, and a lipid profile (Table 1).

After consulting with a pediatric rheumatologist, future plans call for the addition of methotrexate as a steroid-sparing drug in order to further manage the case and reduce steroid reliance.

## Discussion

JDM is a systemic autoimmune vasculopathy marked by muscle weakness and unique dermatological manifestations. Bohan and Peter [17] established the diagnostic criteria for juvenile dermatomyositis. JDM is diagnosed when four of the specified criteria are met, including the skin rash; the diagnosis is considered probable with two more criteria alongside the skin rash. We were unable to do a muscle biopsy, electromyography, or MRI to confirm our clinical assessment in a setting with limited resources. Diagnosing is difficult, particularly in young children, as both electromyography and muscle biopsy are intrusive procedures.

Recently, an international survey among pediatric rheumatologists demonstrated that proximal muscle weakness, specific skin features, and raised muscle enzymes were the most frequently used criteria for identifying JDM; it showed that EMG and muscle biopsy were used as diagnostic support in only 56% and 61% of patients, respectively [18]. Nowadays, diagnosing muscle weakness by MRI is increasingly replacing the latter investigations, but unfortunately, it is not always available in low- and middle-income countries. The combination of skin rash over the face (malar and upper eyelids) and extremities is a common symptom of JDM. JDM was also very likely diagnosed because of the skin nodules and sores on the upper arms (Figure 2), lateral thighs, gluteal region, and abdomen, as well as the weakness, tenderness, wasting, and inability to stand or walk in both lower limbs and high levels of inflammatory markers (ESR) and muscle enzymes (LDH, ALT, and AST).

Diagnosing JDM is challenging, especially in light of the limitation of resources that was reported in a number of studies, such as Kenya (2016) [14], Morocco (2024) [15], Nigeria (2011) [16], and India (2024) [19], where adequate facilities, equipment, and expertise as well as the required lab work may be lacking. The literature identifies muscle weakness and skin manifestations, such as a heliotrope rash and Gottron's papules, as the hallmark features of juvenile dermatomyositis (JDM). Additional symptoms, such as calcinosis and gastrointestinal involvement, may also be present. Our case stands out from atypical JDM presentations, as the patient initially presented with prolonged fever without the characteristic rashes. The skin manifestations associated with JDM appeared only after a month, and no alternative cause for the fever was identified, leading to an initial diagnosis of fever of unknown origin (FUO).

When there are unusual symptoms and high levels of muscle enzymes, JDM should be considered as a possible diagnosis. This has been shown in several studies, including one from Turkey in 2024, which was similar to our case with different initial symptoms; it presented with peri-orbital edema and facial swelling without muscle weakness, and skin findings were reported [20]. Once JDM is diagnosed, emotional support for the family is essential as they confront the uncertainties of a chronic condition. Referrals for counseling can help families adapt to the lifestyle changes associated with caring for children with JDM. Moreover, empowering parents to act as case managers for their child's care is vital. This can be achieved by equipping them with comprehensive knowledge of JDM and the skills needed to navigate the healthcare system effectively. 

In general, JDM management focuses on monitoring the progress of symptoms and normalizing abnormal laboratory values. In this case, inflammatory markers, including elevated erythrocyte sedimentation rate (ESR), triglycerides, and cholesterol, were notable at the time of diagnosis. Therapeutic management was guided by periodic evaluation of laboratory data, the absence of pain, and the maintenance of muscle function.

## Conclusions

To our knowledge, this is the first case was documented in Yemen. The present case illustrates that the atypical and distinctive symptoms of JDM can be easily overlooked, particularly in resource-limited settings without adequate facilities, equipment, and expertise. It shows the difficulty of clinical management and the importance of a multidisciplinary approach in complex patients, especially in settings where diagnostic means are limited. Increasing awareness and understanding of JDM among healthcare providers can enhance diagnostic accuracy and decision-making. While hallmark symptoms such as muscle weakness and skin findings simplify diagnosis, atypical cases require heightened vigilance, as signs may present individually or in varying combinations.
